# Long-term Follow-up Results from PET/CT Surveillance after Surgical Resection of Lung Adenocarcinoma Manifesting as Ground-glass Opacity: Notice of Retraction

**DOI:** 10.1097/01.md.0000482777.50860.38

**Published:** 2016-04-18

The authors have requested that the article “Long-term Follow-up Results from PET/CT Surveillance after Surgical Resection of Lung Adenocarcinoma Manifesting as Ground-glass opacity” be retracted. The authors found several critical errors in the article which were not detected before the publication. Specific details as follows:

1. Substantial number of patients in the study underwent postoperative follow-up with *PET alone* instead of PET/CT. PET/CT was installed in our institution in 2009 and PET (without CT component) was used before 2009. The authors misunderstood that all the patients in this study underwent PET/CT, but recently realized that this was incorrect. *PET alone* may be inferior to PET/CT for detection of small pulmonary lesions due to limited spatial resolution, and the mixed data from PET/CT and *PET alone* might have influenced our study results. The authors’ major concern is that the results from our study
2. Specific details for PET/CT (Methods: Image acquisition, page 2): The specific details regarding the PET/CT was described incorrectly. As stated above, *PET alone* (used before 2009) should have been included in the description. Additionally, the authors made a mistake in describing of the brand name for PET/CT (Gemini, Phillips). The PET/CT scanner used in this study was DVCT, GE healthcare.
3. Considering the mixed data (from *PET alone* and PET/CT), the title of this study (Long-term Follow-up Results from PET/CT Surveillance) is incorrect.

After discussion, the authors concluded that the study may give misinformation to the readers, and therefore they have decided to withdraw the article.
